# Using a WeChat mini-program-based lactation consultant intervention to increase the consumption of mother’s own milk by preterm infants in the neonatal intensive care unit: a study protocol for a cluster randomized controlled trial

**DOI:** 10.1186/s13063-021-05731-6

**Published:** 2021-11-24

**Authors:** Jie Huo, Xinping Wu, Chuanli Gu, Zhangbin Yu, Jun Zhang, Xiaohui Chen, Jingai Zhu, Feng Liu, Beibei Liu, Qianqian Li, Shuping Han

**Affiliations:** 1 Department of Neonatology, Yangzhou Maternity and Child Health Care Hospital, Yangzhou, 225002 China; 2 Department of Neonatology, Nanjing Maternity and Child Health Care Hospital, Nanjing Medical University, Nanjing, 210004 China; 3 Department of Neonatology, Xuzhou Maternity and Child Health Care Hospital, Xuzhou, 221009 China

**Keywords:** WeChat mini-programs, Lactation consultant, Mother’s own milk feeding, Premature infant, Randomized controlled trial

## Abstract

**Background:**

The benefits of mother’s own milk (MOM) for preterm infants have been widely recognized. Many studies have shown that the rate of breastfeeding of premature infants remains very low. Although many studies use measures to promote breastfeeding, few high-quality cluster randomized controlled studies have evaluated the effectiveness of these measures. WeChat is an instant messaging software for smart terminals, and WeChat mini-programs have been widely used to promote health and self-management in China. Based on this background, we designed a randomized controlled study based on WeChat mini-programs to promote MOM feeding of premature infants in the neonatal intensive care unit (NICU).

**Methods/design:**

This study will evaluate the effectiveness of WeChat mini-programs to increase the consumption of MOM feeding in twelve NICUs in Jiangsu Province, namely, six “intervention” NICUs and six “control” NICUs. The study process is as follows: (1) design and preparation, (2) NICU recruitment and training, (3) interpretation and analysis of baseline data, (4) quality control implementation process, and (5) data analysis feedback and publication of study reports. The primary outcome is the proportion of MOM feeding of premature infants during NICU hospitalization. The secondary outcomes are as follows: (1) time to initiation of MOM feeding (hours) and proportion of first-time MOM feeding (%), (2) duration of parenteral nutrition (days), (3) time to total gastrointestinal feeding (days), (4) hospitalization time and hospitalization cost, and (5) incidence of complications (necrotizing enterocolitis, bronchopulmonary dysplasia, feeding intolerance, late-onset sepsis, retinopathy of prematurity).

**Discussion:**

This study is the first cluster randomized controlled trial on the intervention of using a WeChat mini-program-based lactation consultant for premature infants in the NICU in China. We hope this study can improve the consumption of MOM by NICU premature infants during hospitalization through the intervention of WeChat mini-programs.

**Trial registration:**

ClinicalTrials.gov NCT04383379. Registered on May 5, 2020.

**Supplementary Information:**

The online version contains supplementary material available at 10.1186/s13063-021-05731-6.

## Background

The benefits of breastfeeding for preterm infants have been widely recognized, especially mother’s own milk (MOM) feeding, because it can not only reduce the incidence of preterm infant complications, such as retinopathy of prematurity (ROP) [1], necrotizing enterocolitis (NEC) [2, 3], infection [4, 5], and bronchopulmonary dysplasia (BPD) [6], but also improve the development of the nervous system [7] and health status in adulthood [8].

In recent years, with the increasing prevalence of prenatal lactation consultation, breastfeeding education, and guidance, mothers of preterm infants have greatly improved their understanding of breastfeeding worldwide. However, a large gap persists in different regions and neonatal intensive care units (NICUs) for MOM feeding of preterm infants, which may be due to closed management practices at most NICUs in China and the prohibition of parents in the NICU ward. In each NICU, the measures of feeding and other factors are related because they can directly affect the volume of breast milk expression of preterm mothers and whether preterm infants can accept MOM feeding. The American Academy of Paediatrics (AAP) suggests that premature infants should be breastfed as early as possible, and many hospitals have developed strategies to promote breastfeeding. The US Centers for Disease Control and Prevention (CDC) has suggested that breastfeeding should be promoted by professional institutions. MOM is considered the gold standard nutrition for NICU premature infants [9]. A multicentre study [10] reported that in China, only 23% of premature infants in the NICU receive MOM feeding during hospitalization, and this is only partial MOM feeding rather than exclusive MOM feeding. In 2020, an early postpartum breastfeeding rate goal of 75% was set for healthy women [11], but the growth of the breastfeeding rate is very low or even stagnant [12].

Although many studies have been conducted to promote exclusive breastfeeding interventions, few high-quality randomized controlled trials have been performed. Breastfeeding interventions have been demonstrated to be effective in improving the rate of exclusive breastfeeding, especially individualized interventions related to the promotion of breastfeeding [13]. The success of breastfeeding is influenced by socioeconomic, cultural and personal factors (maternal age, disease, delivery mode, premature status), and other factors, which mainly depend on breastfeeding education and support provided by medical professionals [14–16].

In recent years, the number of smartphone users has increased, and some devices will be an important part of health promotion and self-management [17]. Currently, many breastfeeding applications have been used in the clinic to help new mothers understand breastfeeding and provide breastfeeding guidance after discharge [18]. In 2019, an app called “Milk Man” developed in Australia, which can record breastfeeding information and provide support for fathers and expectant fathers to prepare for breastfeeding throughout the perinatal period, showed strong applicability and good effect with game-form forum communication [19]. WeChat is an instant messaging software for smart terminals launched by Tencent on January 21, 2011. It can provide users with chat and WeChat payment capabilities, circles of friends, public platforms, WeChat mini-programs, and other functions. Its users cover more than 200 countries and more than 20 languages. WeChat is widely used by people of different ages, cultural backgrounds, and industries. There are 1.15 billion monthly active users (2019, 11, 3), and it is one of the most popular social platforms in China. More than 300 million WeChat mini-programs are used every month [20]. WeChat mini-programs are applications that can be used without downloading and installation and can meet the needs of different people and industries. In the era of WeChat mini-programs, users only need to scan or search to use them, making these programs convenient and efficient.

Based on this background, we designed a lactation consultant intervention based on the WeChat mini-programs to promote the MOM feeding of premature infants in the NICU. To further improve the quality of the intervention, we have planned a cluster randomized controlled trial. Using the WeChat mini-program as a lactation consultant, we can improve the lactation of premature mothers such that premature infants can receive MOM feeding as soon as possible during hospitalization in the NICU to improve the prognosis of premature infants. If this program is successfully implemented and proven effective, it can be promoted in other NICUs in China.

## Methods/design

### Study objectives

The primary objective of the study is to evaluate the effectiveness of using the “Ning BX breastfeeding” WeChat mini-program lactation consultation intervention for improving the proportion of MOM feeding of premature infants during hospitalization in the NICU.

The secondary objective of this study is to explore the other related effects of the intervention on MOM feeding of premature infants: (1) to shorten the time for premature infants to receive mother’s milk for the first time and increase the proportion of first-time MOM feeding; (2) to shorten the duration of parenteral nutrition and achieve full enteral gastrointestinal feeding as soon as possible; (3) to shorten the hospitalization time and reduce the hospitalization cost; and (4) to reduce the incidence of premature complications, such as necrotizing enterocolitis (NEC), bronchopulmonary dysplasia (BPD), late-onset sepsis (LOS), and retinopathy of prematurity (ROP).

### Study design

**Fig. 1:**
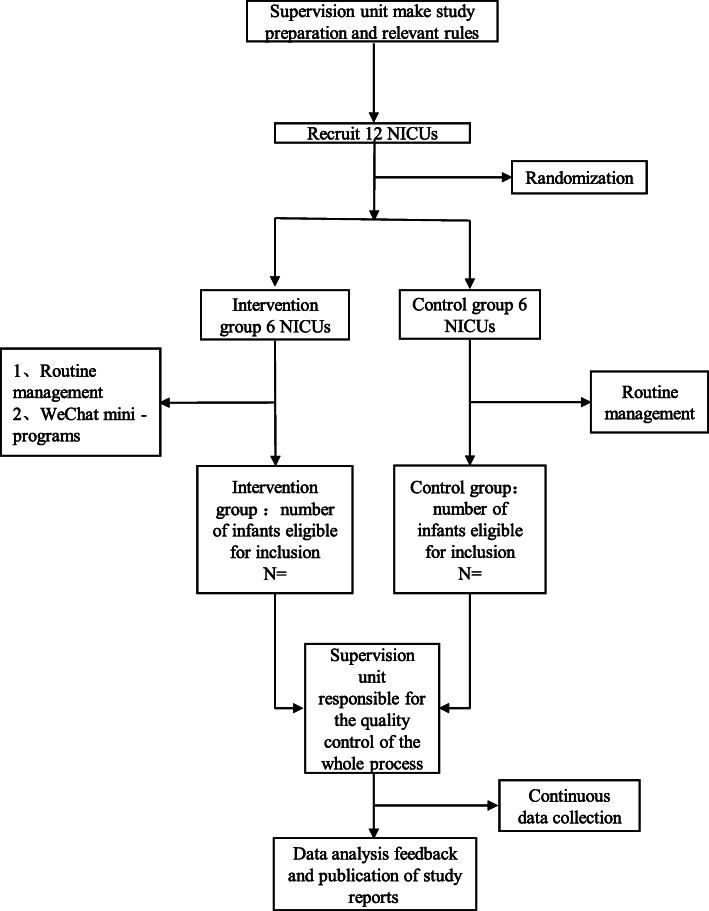
Study protocol flowchart of MOM feeding lactation consultant intervention via WeChat mini-programs

**Fig. 2:**
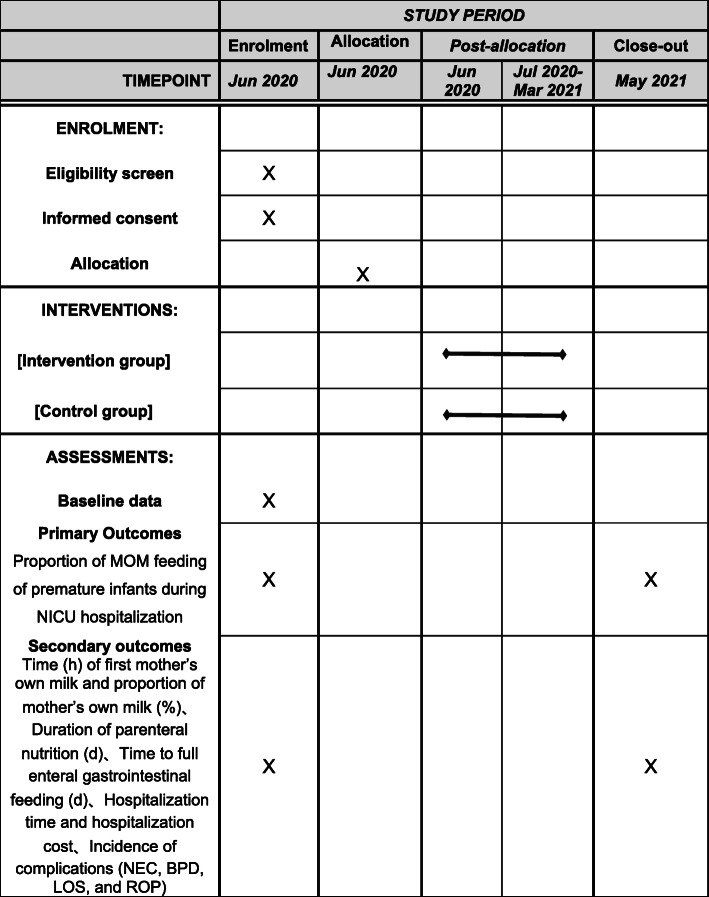
Schedule of enrolment, interventions, and assessments

In this study, a cluster randomized controlled matching design will be used. This protocol follows the 2013 SPIRIT guidelines (Additional file 1), and the hospital has been chosen as a cluster. The supervision unit will be used for quality control, coordination and technical support during the entire study. In this study, we will recruit 12 NICUs. The two NICUs with a similar number of premature infants, the same NICU scale, and the same range of MOM feeding proportion taken as a floor will then randomly be divided into two groups, namely, an intervention group of 6 NICUs and a control group of 6 NICUs. In the intervention group, only the guardian of premature infants used the “Ning BX breastfeeding” WeChat mini-programs, and other treatments were the same as those in the control group. The guardian could choose to withdraw at any time, which does not affect any treatment of premature infants. The control group will receive only routine management. Flowcharts of the study design and timeline are provided in Figs. 1 and 2, respectively.

### Hypothesis

We hypothesize that if the “Ning BX breastfeeding” WeChat mini-programs are implemented successfully, the proportion of MOM feeding of premature infants during hospitalization in the NICU will increase by at least 15%; the second hypothesis is that the length of hospitalization and medical expenses will be reduced, and the quality of life of the premature infants will be improved.

### Eligibility and exclusion criteria

#### Inclusion criteria

1. Preterm infants weighing less than 2000 g and/or with a gestational age of less than 34 weeks.
2. The guardians of all preterm infants will provide informed consent.

#### Exclusion criteria

1. Pregnancy with serious diseases (such as malignant tumour, epilepsy or heart dysfunction), infectious diseases, or other medical contraindications for breastfeeding (such as galactosemia);
2. No parental consent.

### Randomization

Because the intervention cannot be completely blinded in this study due to the randomization of groups as units and differences in NICU scale, a whole-group matching design will be adopted. Twelve units will be randomized into two groups (intervention and control groups) instead of patient randomization to avoid the confounding effect of patients in the control groups. Two NICUs with a similar number of premature infants, the same NICU scale, and approximately the same range of MOM feeding taken as a floor will be randomly divided into six groups. Next, the names of 12 units will be written on paper and placed in the same envelope. One third-party individual blinded to this project will take one envelope from each of the six groups, and the six chosen envelopes will be assigned to the intervention group. The remaining six envelopes will be assigned to the control group.

### Sample size calculation

The proposed sample size is based on the primary outcome of the proportion of MOM feeding. According to the baseline data reported (unpublished data) by each unit in 2019, the average proportion of MOM feeding was 42.46%. We expect the proportion of MOM feeding of premature infants during NICU hospitalization to increase by at least 15% in the intervention group compared with that in the control group. We will achieve 90% power to detect a difference between the group means of at least 6, and the standard deviation of subjects is 30.00, assuming the intracluster correlation coefficient is 0.005 [21, 22]. We estimate a total sample size of 1620 (810 in the intervention group and an average of 135 in each group). Considering a 10% drop-out rate following enrolment in the study, we will enrol 900 premature infants in the intervention and control groups. To achieve this sample size, we will conduct a 1-year study in 12 NICUs.

### Intervention

**Fig. 3:**
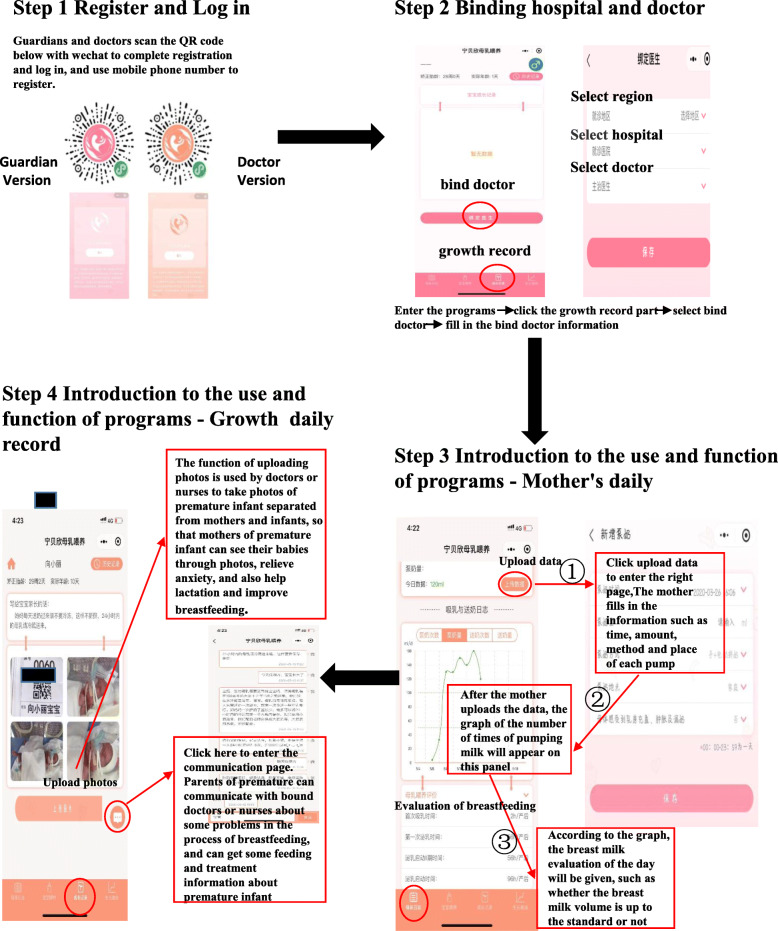
Function of instructions for the “Ning BX breastfeeding” WeChat mini-programs

In 2019, the “NingBX neonatal homogeneity platform” team developed a WeChat mini-programs named “Ning BX breastfeeding”, which is used to address the problems related to breastfeeding and lactation consultation of premature infants and mothers with NICU mother-infant separation. The “Ning BX breastfeeding” WeChat mini-programs have been in the trial phase at Nanjing Maternity and Child Health Care Hospital, with very promising results. The “Ning BX breastfeeding” WeChat mini-programs are divided into the doctor version and guardian version. By scanning the corresponding QR code, a mobile number can be entered and used to register and log in. A mobile number can be registered only once to protect the privacy of users and prevent information leakage. The WeChat mini-programs educators will be responsible for obtaining informed consent from the guardians of premature infants. Informed consent was provided by the guardians of all participants, who could be withdrawn at any time according to the wishes of their guardians without affecting any treatment. By binding the corresponding hospital and designating the doctor, information can be shared. The “Ning BX breastfeeding” WeChat mini-programs is divided into four parts (Fig. 3): Mother’s Daily, Baby Feeding, Growth Record, and Growth Curve.

### Part 1 Mother’s Daily

The mother’s daily lactation information, such as the number of pumps, volume of feeding by pumping, number of feeding sessions, and volume of feeding, is recorded. The mother can record the time, place, mode, and volume of each pump in detail, and a curve is constructed between the number and volume of pumps. By continuously uploading information from the mother, a breastfeeding evaluation can be automatically performed to assess information such as first lactation time, the start time of lactation phase II, and the start time of lactation, which are divided into three levels: good, average, and poor. This feature can record the entire process of a mother’s lactation, which is different from the traditional paper diary or recall recording of the pumped milk information. It can assess lactation information in a more timely and accurate manner and can provide professional guidance and intervention for mothers who have difficulty starting lactation in a timely manner.

### Part 2 Baby Feeding

Baby feeding is used to record the daily feeding information of premature infants discharged from the NICU after returning home. The mother can upload the time, volume, type (mother’s own milk, amino acid formula, ordinary formula), and daily weight of the infant. The program can evaluate whether the infant is fed adequately and whether the weight growth is within the normal range according to the amount and weight of the infant to guide the mothers of premature infants on whether professional outpatient consultation is needed. Because this study will focus on premature infants during hospitalization, this section is not currently available.

### Part 3 Growth Record

The growth record is used to interact with mothers of preterm infants admitted to the NICU. Doctors or nurses can upload photographs of babies in the NICU who are separated from their mothers and other infants to inform the mothers about the current situation and to promote earlier lactation by mothers. Additionally, this feature also has the function of communication. The mothers of premature infants can communicate with their assigned doctor, ask about the current diagnosis and treatment of the baby and feeding situation, assess the difficulties and challenges encountered in the process of breastfeeding, and obtain responses to questions from professional personnel.

### Part 4 Growth Curve

A growth curve is used to record the growth indicators of premature infants, such as weight, length, and head circumference, and carry out nutritional risk assessment and feeding guidance using this information. The mother can regularly upload the baby’s weight, length, and head circumference records, automatically generate the Fenton growth curve, and carry out nutrition risk assessment to determine whether the baby’s growth and development are normal. Additionally, the growth curve can provide feeding guidance or suggestions for the growth and development clinic according to the assessment results. This section is currently not available.

### Pre intervention training

Before the study, the supervision unit will be responsible for the training on the use of the “Ning BX breastfeeding” WeChat mini-programs and the development of a user guide. The intervention group unit will establish a “Ning BX breastfeeding” WeChat mini-programs implementation team comprising at least one neonatologist and one neonatal nurse. Each participant needs to be trained in the use of the “Ning BX breastfeeding” WeChat mini-programs and master the ability to teach parents how to use it. Each intervention group unit will formulate a corresponding implementation process based on the actual management situation of the unit. The supervision unit will set up a “Ning BX breastfeeding” WeChat mini-programs communication group to communicate difficulties and doubts encountered in the implementation process over time and provide help to some units encountering relatively difficult implementation.

### Quality control of implementation process

The supervision unit is responsible for the quality control, technical support, and experience transfer of the entire implementation process and is assigned by a dedicated person. The unit includes the following aspects: (1) the supervision unit regularly assigns professionals to the site to provide guidance and propose problems and solutions; (2) each participating unit surveys the user regarding his/her experience with the mini-programs, initially and during and after use if needed; (3) the “Ning BX breastfeeding” WeChat mini-programs data will be exported every month for data quality control, assessment of feedback problems, and provision of timely updates; and (4) a face-to-face session to assess and compare the doctor user and guardian user versions will be held to promote the best use of the WeChat mini-programs. ⑤If adverse events occur in the process of implementation, the participating units shall collecting and report to the supervision unit. Finally, the ethics committee make a decision, and the supervision unit shall inform the participating units of the results.

### Control group

The control group will be given routine management according to the existing management measures and policies of the unit, such as being provided with traditional education and the task of completing a breast milk paper diary. The control unit will not use the “Ning BX breastfeeding” WeChat mini-programs.

### Data collection

The data collection in this study will be performed according to the online database “NingBX neonatal homogeneity platform” (https://www.ningbx.com/) developed by a collaboration group in 2019. The basic data required for this study, such as the birth time, gestational age, weight, diagnosis and treatment process, complications, outcome, and other information, will all be obtained from the online database. The data related to breastfeeding will also be obtained from online databases, such as MOM at the time of feed initiation (hours), proportion of first-time MOM (%), duration of parenteral nutrition (days), time to total gastrointestinal feeding (days), total volume of breastfeeding per day, and total volume of breastfeeding during hospitalization. All the data in the online database will be input by professional personnel who are trained and qualified for entry. The database quality control personnel regularly control and feedback the data, and the database can modify and update the information in real time, as well as export the data in real time for data quality control and statistical analysis. Some of the database content is provided in Additional file 2.

### Data management

Nanjing Maternity and Child Health Care Hospital, as the supervision unit, will formulate the database entry guide and provide a detailed definition of each variable in the database to ensure the consistency of data entry. The data entry personnel shall be doctors or postgraduates with rich clinical experience in neonatology, and the chief physician of neonatology or personnel with a higher professional title shall be data quality control personnel. The supervision unit is responsible for the selection, training and assessment of the input, as well as for quality control personnel in the cooperation group. Only those who regularly participate in the training and pass the assessment can participate in the data entry work and become database entry personnel. Additionally, the supervision unit will be responsible for regular training, mid-term assessment and year-end summary of data entry throughout the entire research period. The supervision unit will hold an online meeting for the quality controller and recorder every month to discuss the problems encountered with the recent input and quality control data, rectification plan, and experience sharing. The professional personnel in charge of the database management of the supervision unit will conduct unified quality control for all data, generate a summary, and provide feedback on the opinions to all participating units in a timely manner. Statistical experts will analyse the data every 3 months, summarize the statistical results, and recheck and update the ambiguous results. In addition, the supervision unit as the coordination centre is responsible for analysing and publishing the data in an anonymized form. The data monitoring committee consists of the data quality control personnel of the coordination centre and chief investigators of 12 participating NICUs, and it will be responsible for monitoring the conduct of the study to ensure compliance with the study protocol. The committee is also responsible for other aspects, including providing study materials, checking and analysing data, and publishing study reports (if there are any changes to the protocol, the new study report through email and WeChat mini-programs inform 12 participating NICUs). The ethics committee, which is independent of the sponsor and investigators, will audit the conduct of the study annually.

### Data association

The doctor and guardian versions of the data in the WeChat mini-programs can be shared and stored in an online database. The online database has a special data export function for the “Ning BX breastfeeding” WeChat mini-programs. The three functions form a closed loop, which can realize the timely export and sharing of the data (Fig. 3).

### Statistical analysis

Statistical analysis will be performed by statisticians; continuous variables will be presented as the means and standard deviations, and categorical variables will be presented as numbers and proportions. Adjusted chi-square analysis will be performed to compare the primary outcome indicators of the intervention and control groups, and the cluster effect will be considered. The two-level stratified logistic regression model will be used to compare the main outcome indicators between the two groups.

Superiority test will be used if necessary. The results will be adjusted based on an evaluation of neonatal characteristics (such as gestational age, small for gestational age, gender, and 5-min Apgar score) and NICU characteristics (number of preterm infants admitted and rate of MOM feeding). All statistical tests will be two-tailed, and *P* < 0.05 will be considered significant.

### Outcome and definitions

The primary outcome is the proportion of MOM feeding of premature infants during NICU hospitalization.

The secondary outcomes are as follows (Table 1): (1) MOM at feeding initiation (hours) and proportion of first-time use of MOM (%); (2) duration of parenteral nutrition (days); (3) time to total gastrointestinal feeding (days); (4) hospitalization time and cost; and (5) incidence of complications, such as NEC, BPD, LOS, and ROP.

**Table 1:** Outcome and definitions

Categories		Source of data	Implementation process control	Definitions
**Primary outcomes**	Proportion of MOM feeding of premature infants during NICU hospitalization [23]	Online database	Continuous data entry of each unit regular data export for statistics	Calculated to determine the relative amount of MOM and total milk (human milk+formula, TM) taken by each infant during NICU hospitalization [mLsMOM/mLsTM]*100
**Secondary outcomes**	Time (h) of first mother’s own milk and proportion of mother’s own milk (%)	Online database	Continuous data entry of each unit regular data export for statistics	Time after birth to the first MOM and proportion of the first MOM to the total milk
	Duration of parenteral nutrition (d)	Online database		Duration of total parenteral nutrition required for complete fasting in preterm infants
	Time to full enteral gastrointestinal feeding (d)	Online database		Full enteral gastrointestinal feeding is defined as no longer in need of parenteral nutrition or fluid
	Hospitalization time and cost	Online database		Hospitalization time is the time in the NICU, and hospitalization cost is the total cost during the hospital stay
	Incidence of complications (NEC, BPD, LOS, and ROP )[24]	Online database		Incidence of common complications in preterm infants, such as necrotizing enterocolitis (NEC), bronchopulmonary dysplasia (BPD), late-onset sepsis (LOS), and retinopathy of prematurity (ROP)

## Discussion

This is the first cluster randomized controlled trial in China in which WeChat mini-programs will be used to improve MOM feeding during the hospitalization of premature infants. The goal of this study is to increase the proportion of NICU premature infants receiving MOM during hospitalization through the intervention of WeChat mini-programs. If the implementation of the program is successful, it can be promoted in other NICUs.

Breastfeeding for mothers of NICU preterm infants is associated with more challenges than that for mothers of full-term infants. Foreign studies have shown that the rate of MOM feeding of premature infants in the NICU is 76% [25]. However, a multicentre study in China revealed that the rate of exclusive MOM feeding of premature infants during hospitalization was only 20% [26]. Such a large gap is related not only to the management policy of the NICU in China but also to the anxiety and negative emotions of mothers and their families of premature infants and, more importantly, to the lack of effective lactation counselling interventions. WeChat mini-programs can help mothers of preterm infants better participate in the medical process, enhance the emotional communication between mothers and infants, and help prolong breastfeeding. During the novel coronavirus (COVID-19) epidemic period, the communication function of WeChat mini-programs played a significant role. Family members and society should be encouraged to provide more support to pregnant women to improve their confidence in breastfeeding. The purpose of this study is to evaluate the effect of WeChat mini-programs lactation consultation on increasing the proportion of MOM feeding in premature infants in the NICU. We assume that the proportion of MOM feeding in the intervention group will be much higher than that in the control group, and this project can be carried out for a long time.

Our study will have several limitations. The participating units of this study are distributed throughout all parts of Jiangsu Province. Although they can represent the situation of all regions, there may be some regional biases due to the different management modes of NICUs. Therefore, in the early stage of this study, we will obtain baseline data to account for differences due to different NICU policies. Considering that this study is randomized to different units, some patient potential confounding factors, such as gestational age, birth weight, and treatment level, cannot be completely controlled. To reduce the possible deviation between the two groups, multilevel models will be used to compare the primary outcome indicators of the two groups, and these potential confounding factors will be adjusted in the analysis stage.

The goal of our study is to improve the MOM feeding of premature infants during NICU hospitalization using WeChat mini-programs to provide lactation consultation intervention and evaluate the effectiveness of WeChat mini-programs so that it can be promoted nationwide in the future and more premature infants can benefit from it.

## Trial status

The study began in June 2020, and patient registration started in June 2020. The internal pilot study will be completed by the end of June 2020. This research is in the main research stage and was officially registered and completed in March 2021. The entire project will be completed in May 2021. Protocol version number: 1.2.

## Supplementary Information

**Additional file 1.**

**Additional file 2.**

**Additional file 3.**

## Data Availability

The baseline data and data analysed in the current study are available from the corresponding authors upon reasonable request.

## Abbreviations

NICU: Neonatal intensive care unit
MOM: Mother’s own milk
PDSA: Plan-do-study-act
NEC: Necrotizing enterocolitis
BPD: Bronchopulmonary dysplasia
LOS: Late-onset sepsis
ROP: Retinopathy of prematurity
AAP: American Academy Paediatrics
CDC: Centers for Disease Control and Prevention
