# Supplementary material for: Using a WeChat mini-program-based lactation consultant intervention to increase the consumption of mother’s own milk by preterm infants in the neonatal intensive care unit: a study protocol for a cluster randomized controlled trial
Source: Trials. 2021 Nov 24;22:834. doi: 10.1186/s13063-021-05731-6 (PMC8611400; doi:10.1186/s13063-021-05731-6)
Supplement: Supplementary file 2 — Additional file 2. [file 13063_2021_5731_MOESM2_ESM.pdf]

Functions and instructions of online database and wechat applet

online database → NingBX neonatal homogeneity platform

Step 1 This is an online database（https://www.ningbx.com/）, each participating unit has a unique identification user name and password, which is used to collect all information during the hospitalization of children, including data collection, data analysis, data export and other functions

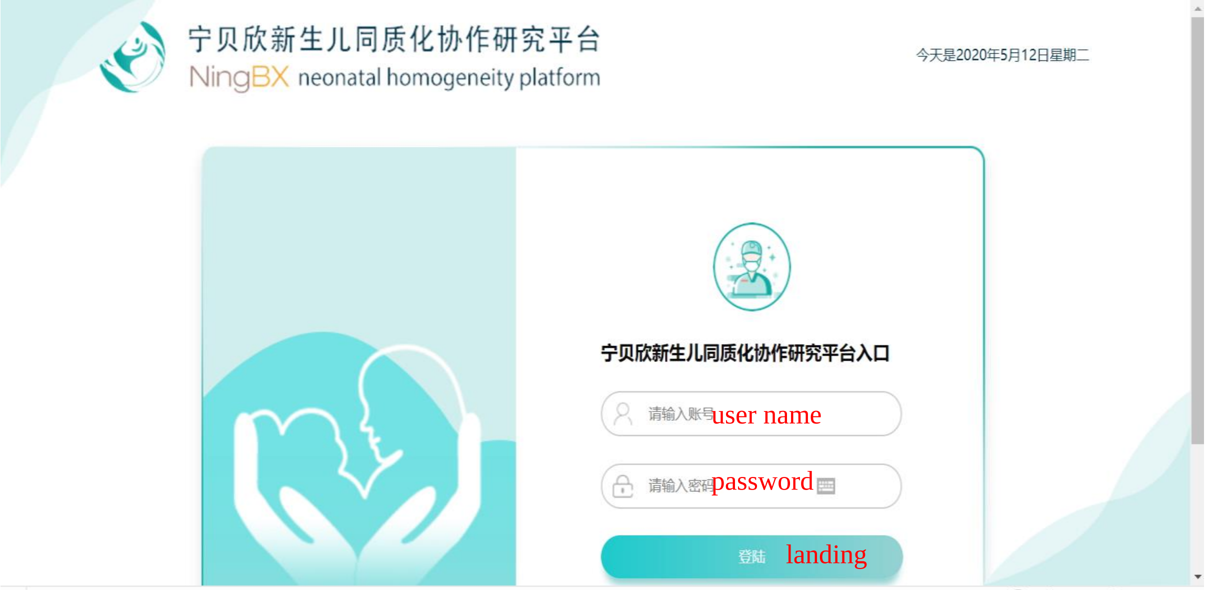

Step 2 The database includes neonatal platform and hyperbilirubinemia platform. In this study, the neonatal platform is used, including 10 parts.

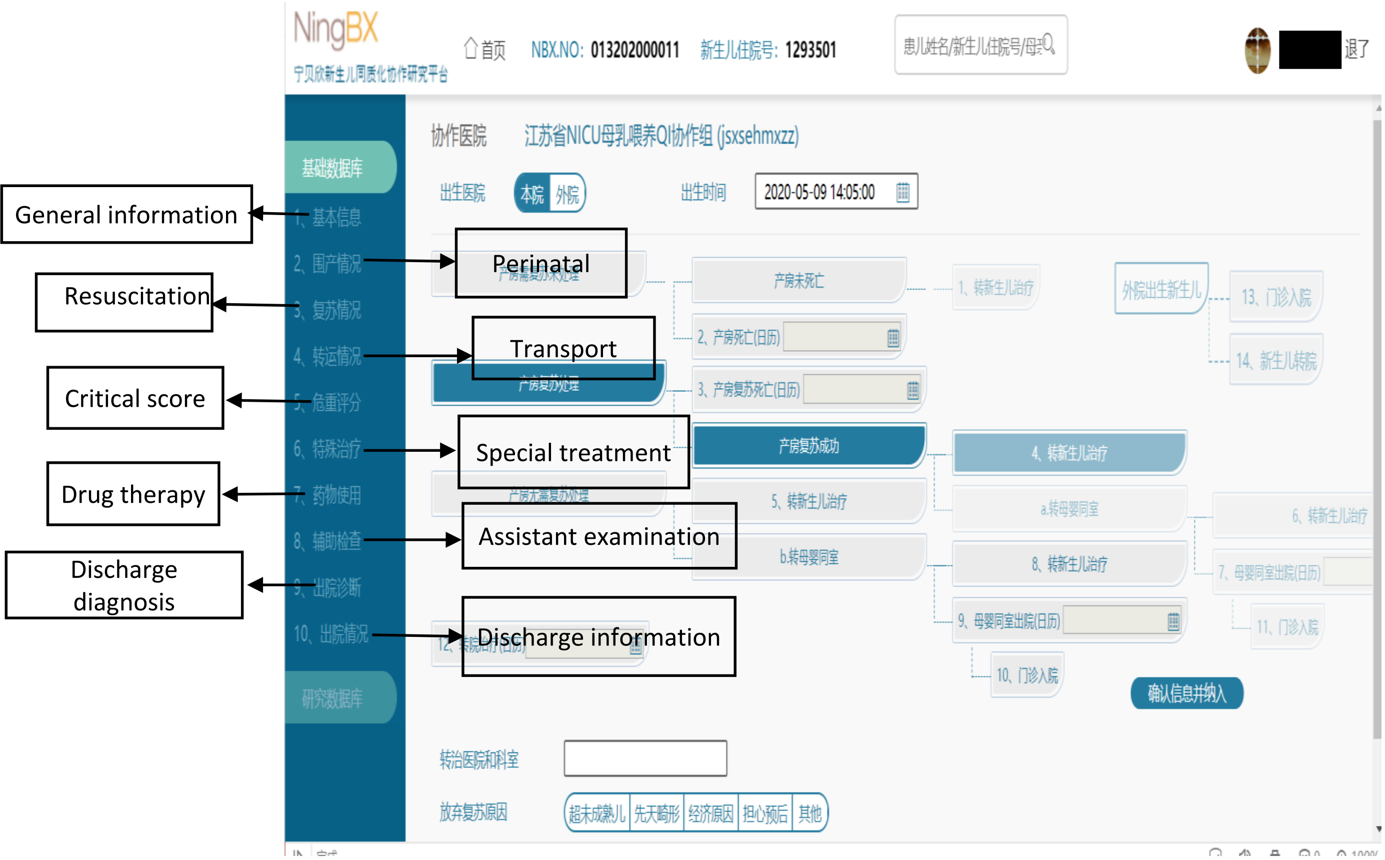

Step 3

Part I General information

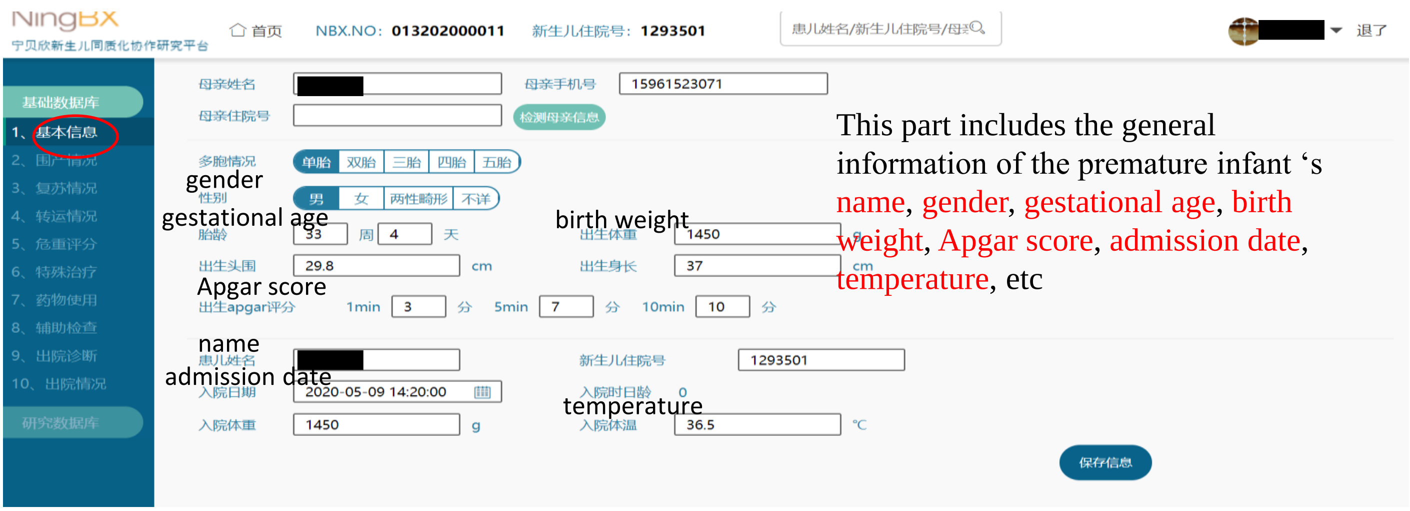

Part 2 Perinatal

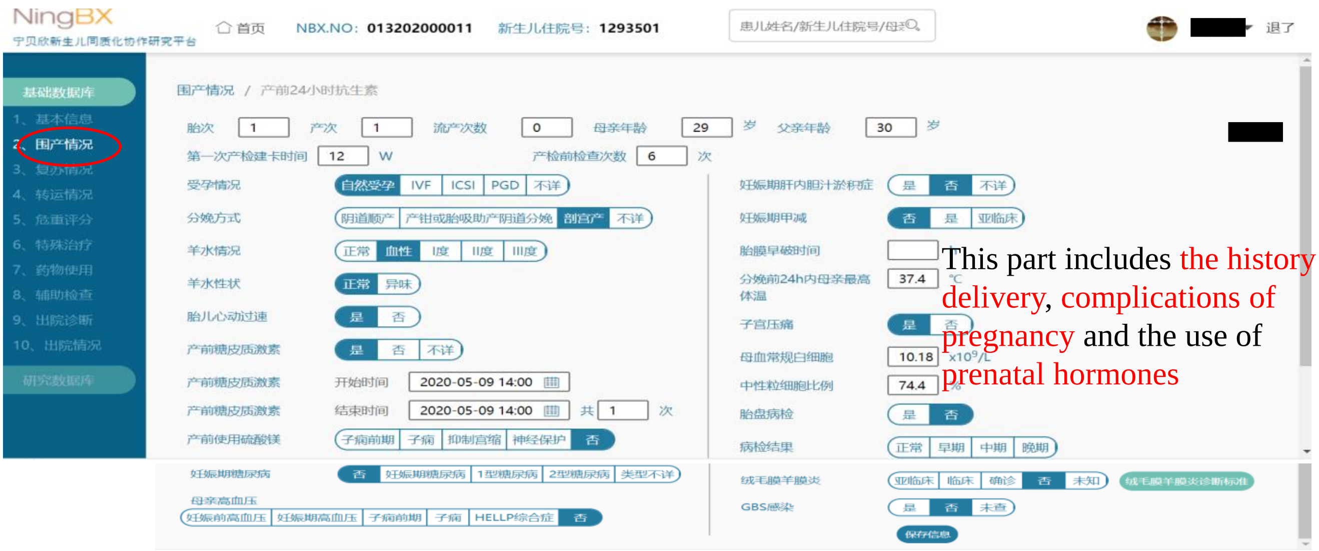

Part 3 Resuscitation

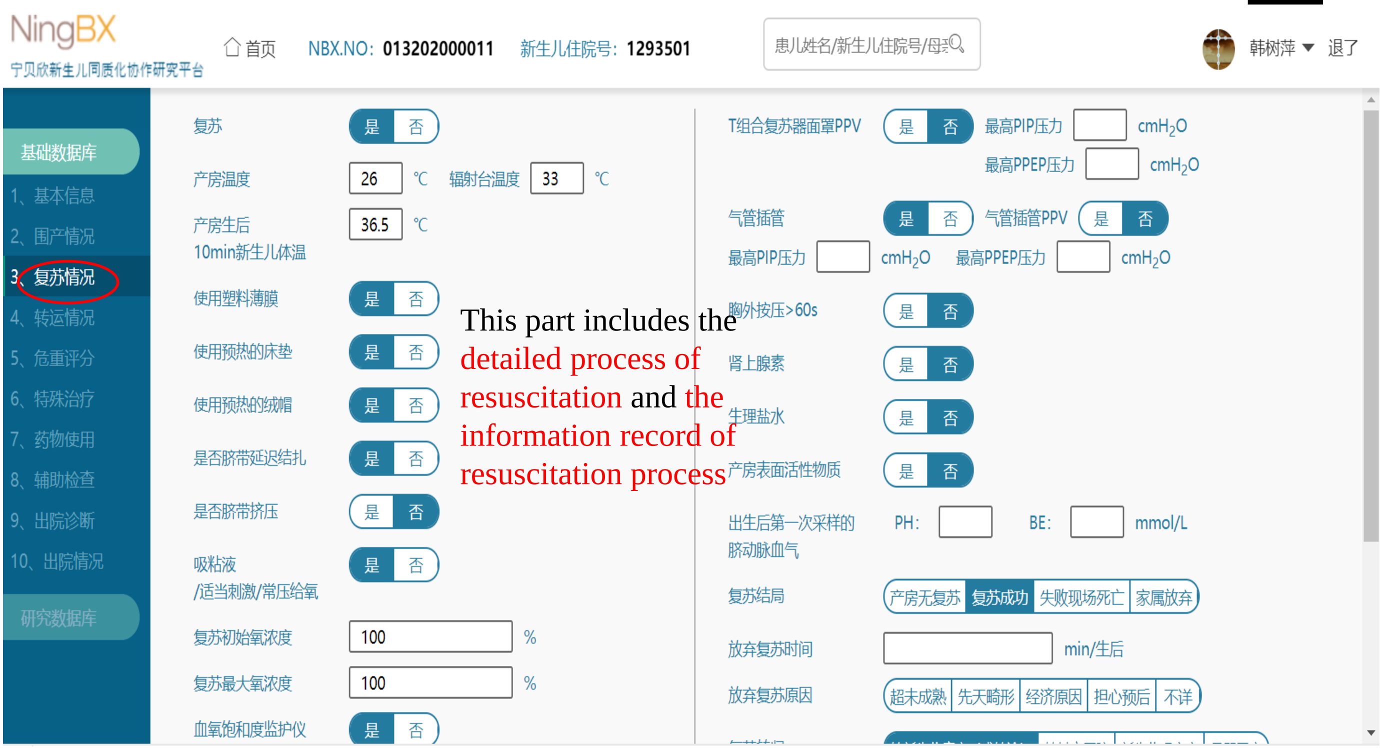

Part 4 Transport

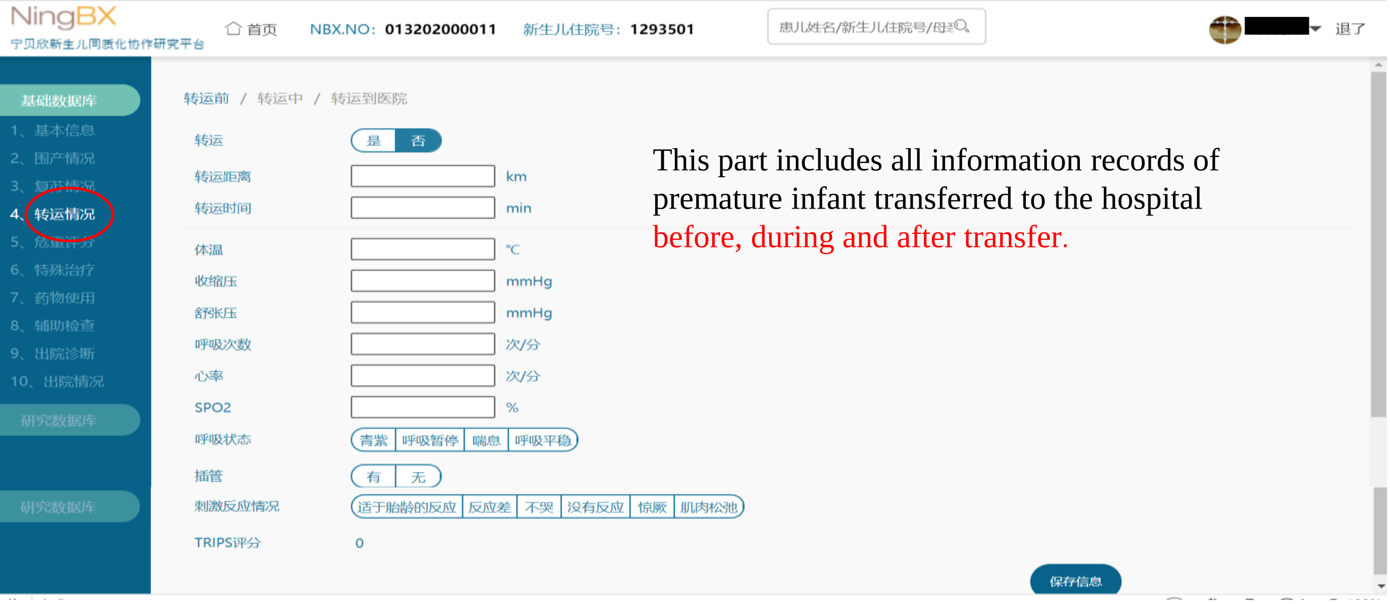

This part includes all information records of premature infant transferred to the hospital before, during and after transfer.

Part 5 Critical score

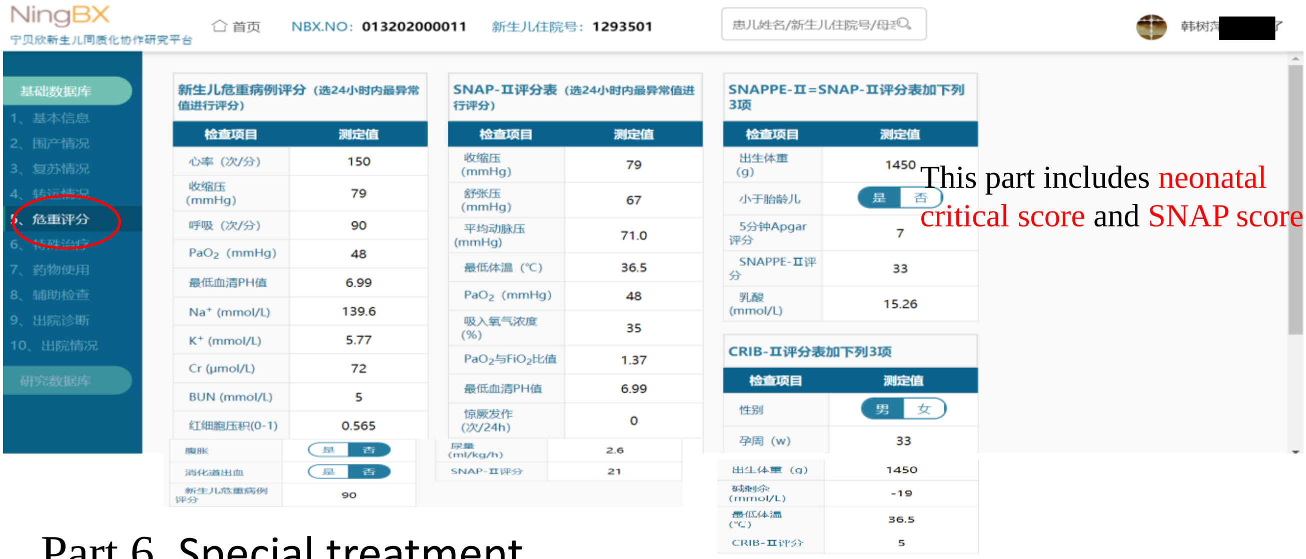

This part includes neonatal critical score and SNAP score

Part 6 Special treatment

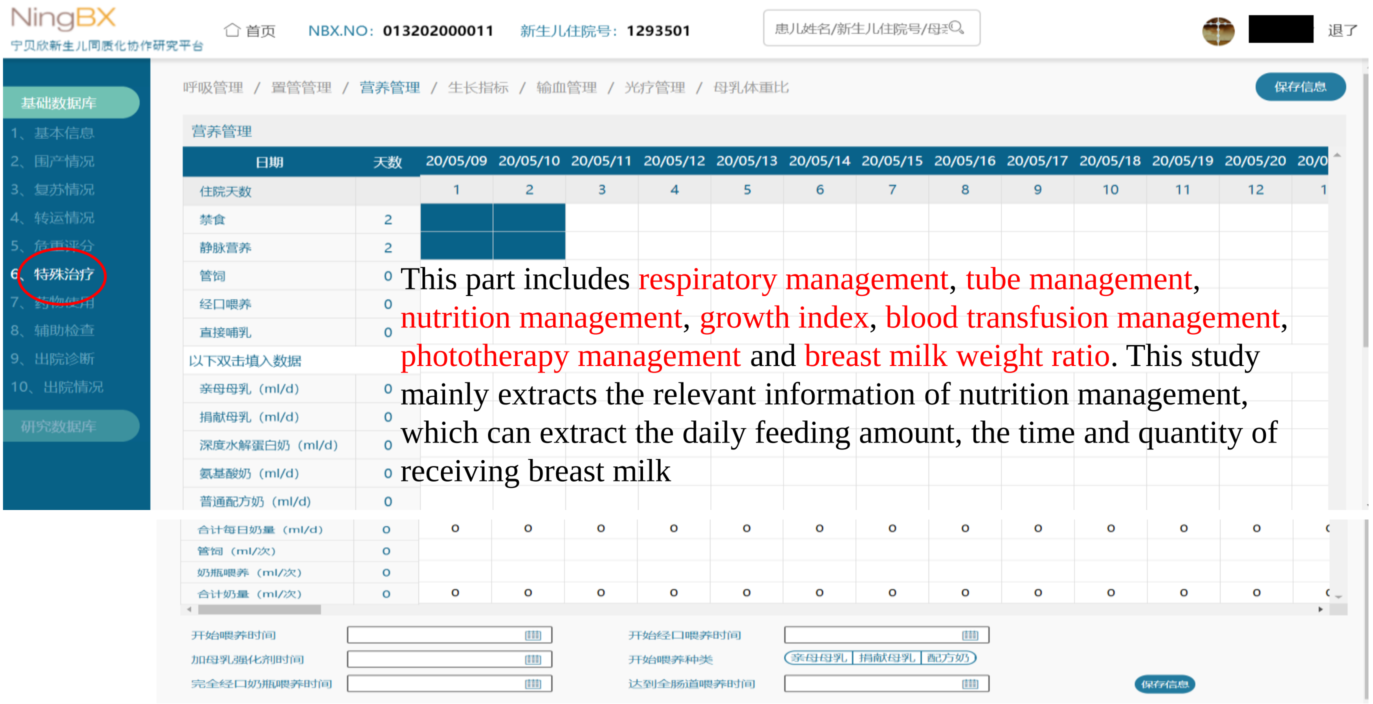

This part includes respiratory management, tube management, nutrition management, growth index, blood transfusion management, phototherapy management and breast milk weight ratio. This study mainly extracts the relevant information of nutrition management, which can extract the daily feeding amount, the time and quantity of receiving breast milk

Part7 Drug therapy

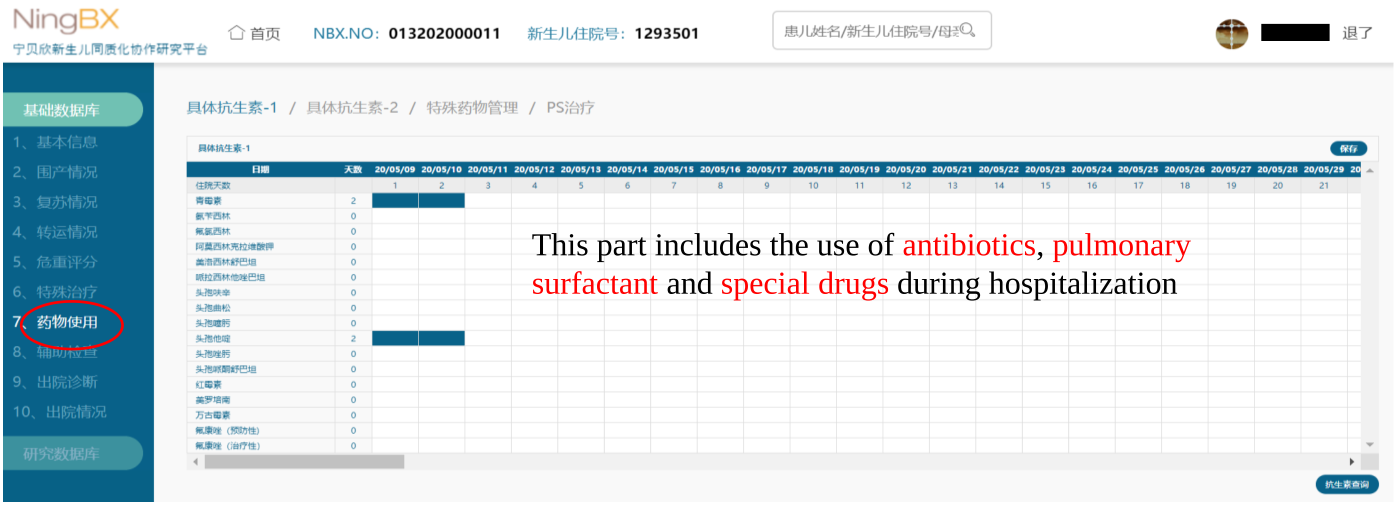

This part includes the use of antibiotics, pulmonary surfactant and special drugs during hospitalization

Part 8 Assistant examination

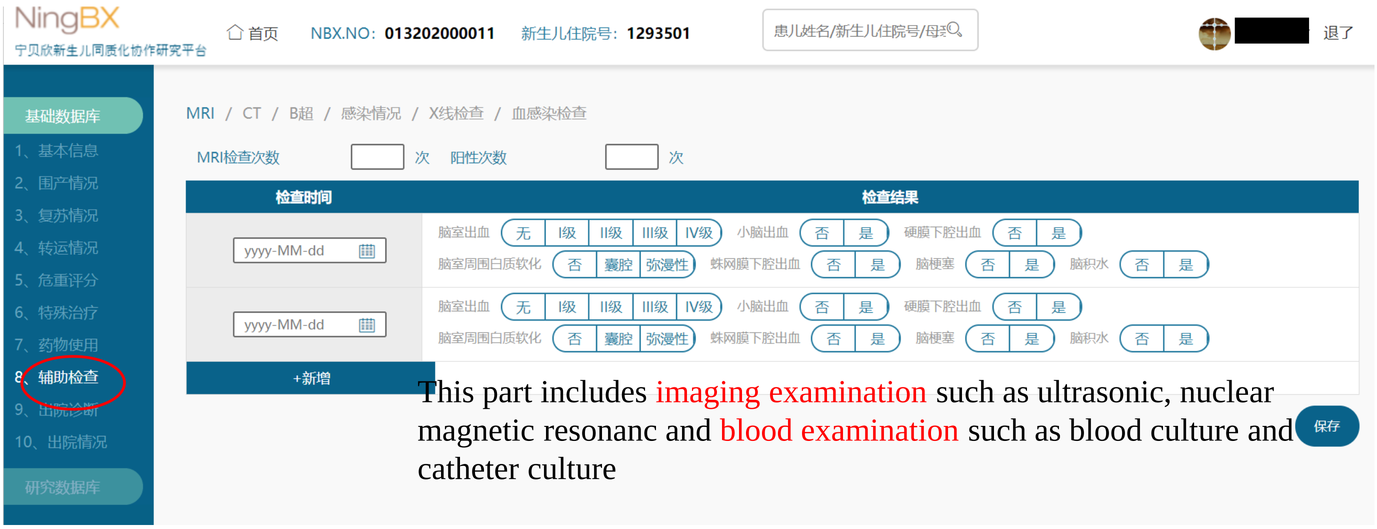

This part includes imaging examination such as ultrasonic, nuclear magnetic resonanc and blood examination such as blood culture and catheter culture

Part 9 Discharge diagnosis

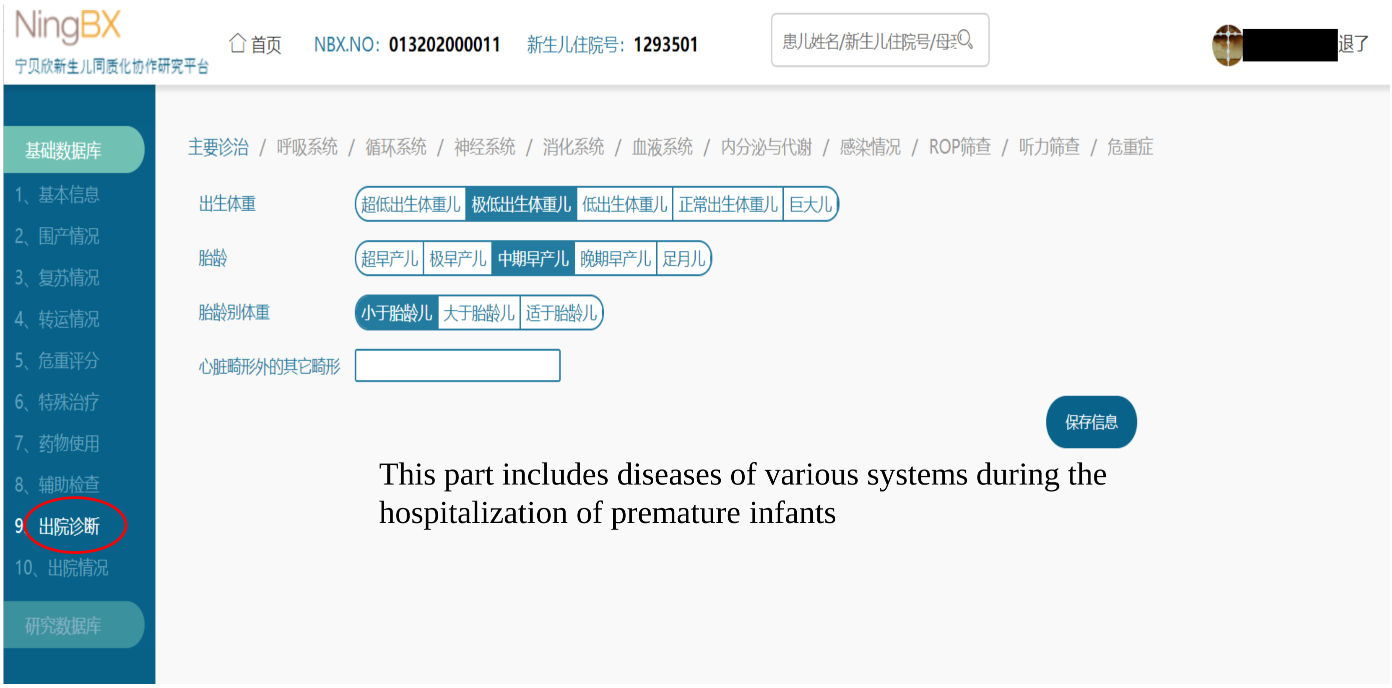

This part includes diseases of various systems during the hospitalization of premature infants

Part 10 Discharge information

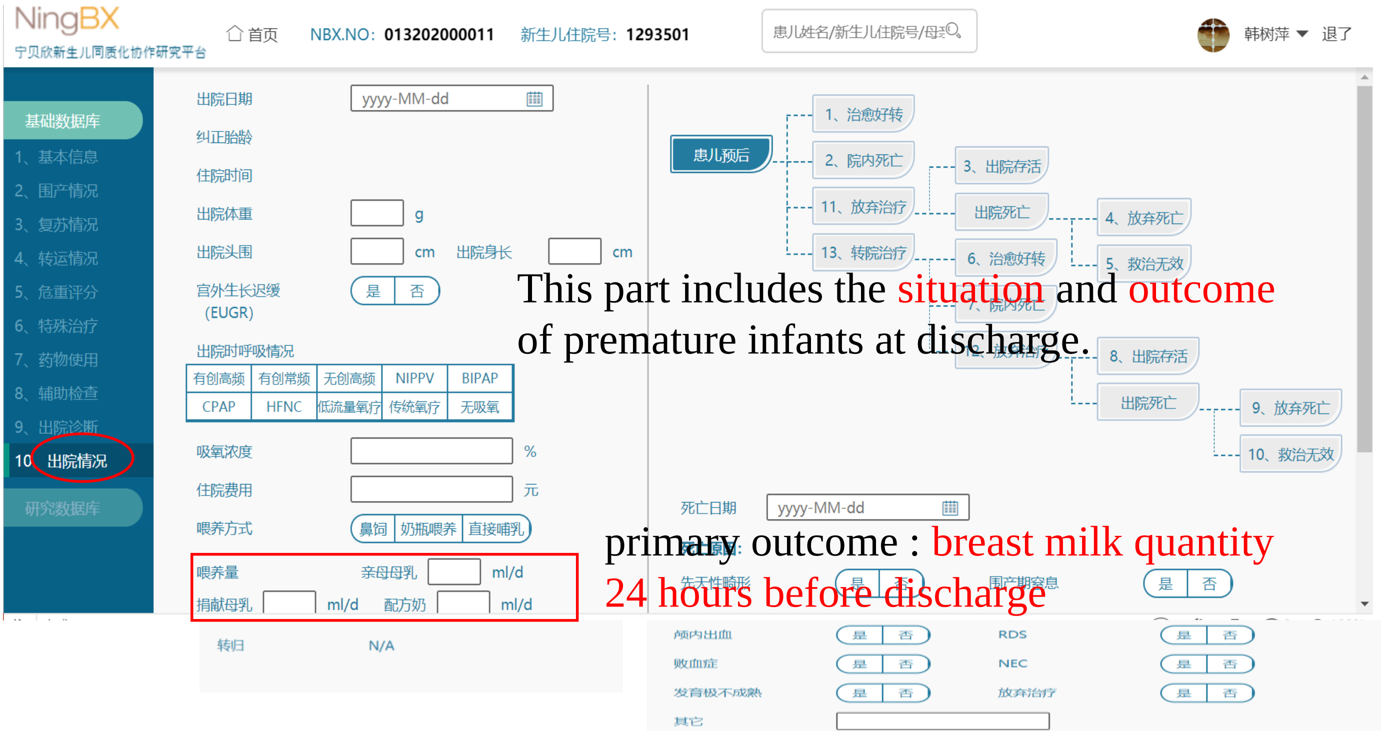

This part includes the situation and outcome of premature infants at discharge.

primary outcome : breast milk quantity 24 hours before discharge

WeChat mini –programs → Ning BX breastfeeding

WeChat mini –programs used to collect information about mother's lactation, pump milk and communicate with mothers of premature infants about breastfeeding.

Step 1 Register and Log in

Guardians and doctors scan the QR code below with wechat to complete registration and log in, and use mobile phone number to register.

Guardian Version

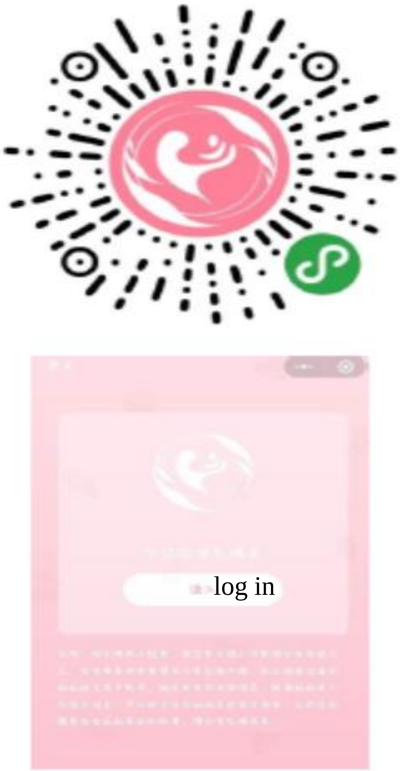

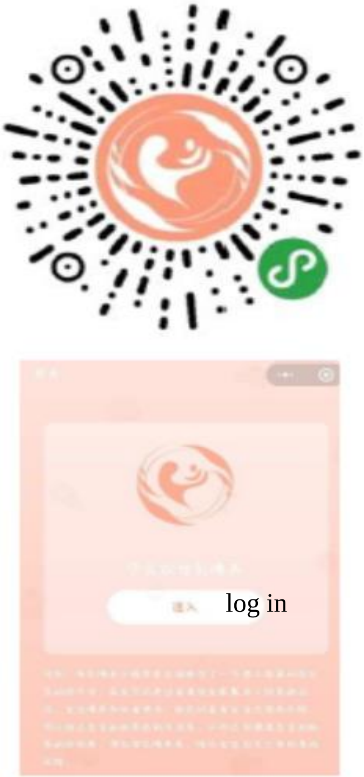

Doctor Version

Step 2 Binding hospital and doctor

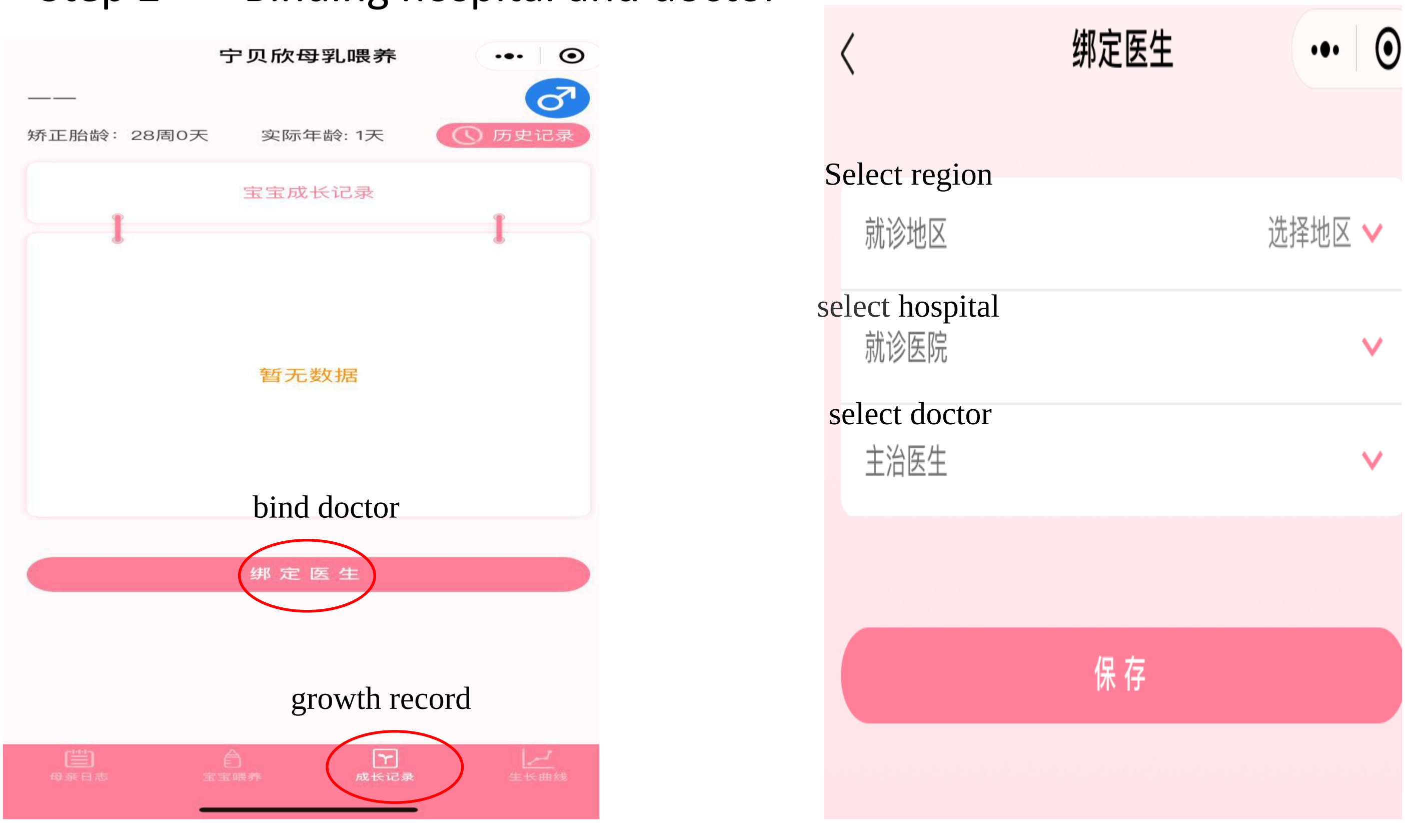

Enter the programs→click the growth record part→select bind doctor→fill in the bind doctor information

Step 3 Introduction to the use and function of programs - Mother's daily

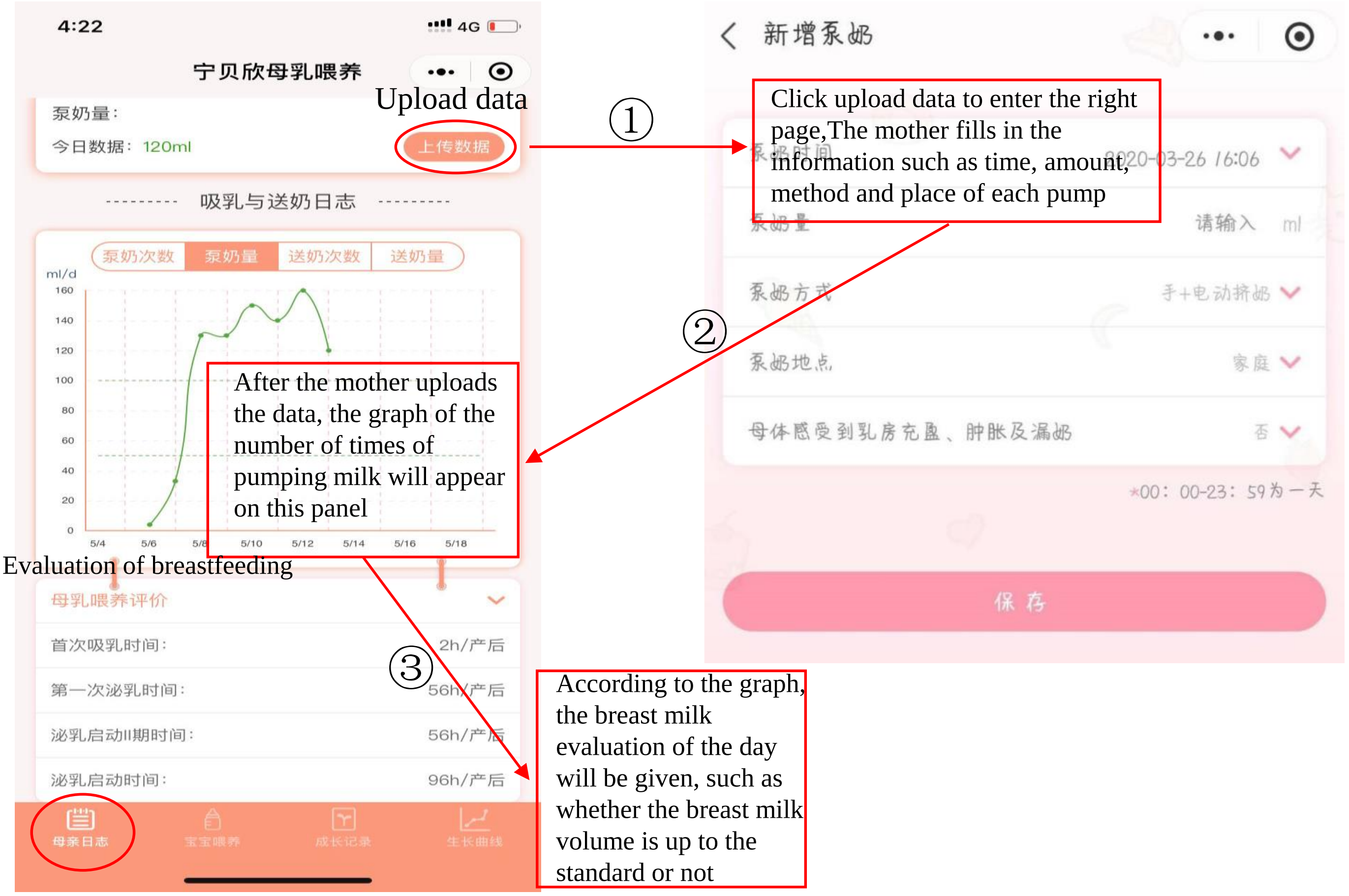

Step 4 Introduction to the use and function of programs - Growth daily record

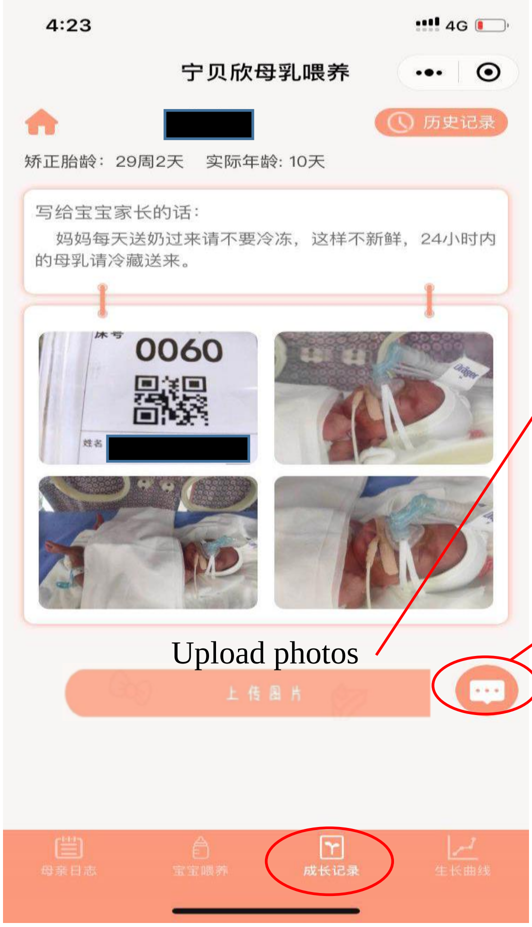

The function of uploading photos is used by doctors or nurses to take photos of premature infant separated from mothers and infants, so that mothers of premature infant can see their babies through photos, relieve anxiety, and also help lactation and improve breastfeeding.

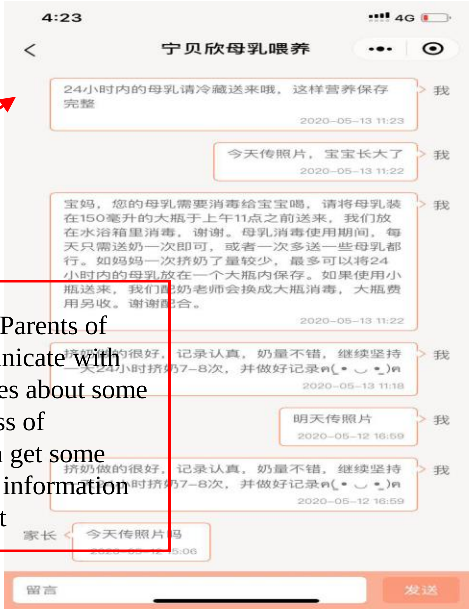

Upload photos

Click here to enter the communication page. Parents of premature can communicate with bound doctors or nurses about some problems in the process of breastfeeding, and can get some feeding and treatment information about premature infant

Step 5 Data association and export

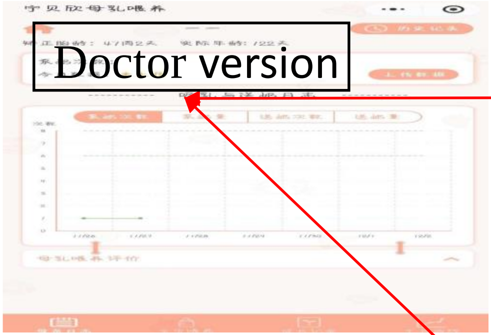

Doctor version

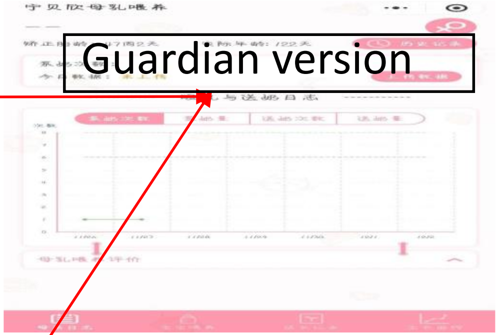

Guardian version

The three forms a closed loop. The doctor version and the guardianl version of the Wechat mini-programs share the data, and all the information is stored in the online database, which can realize the timely export and sharing of the data.

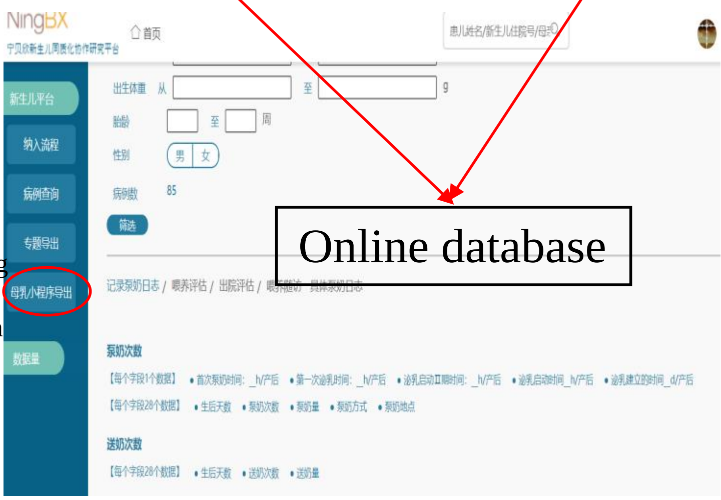

Breastfeeding applet data export button

Online database
