# Supplementary figures and images for: Using a WeChat mini-program-based lactation consultant intervention to increase the consumption of mother’s own milk by preterm infants in the neonatal intensive care unit: a study protocol for a cluster randomized controlled trial
Source: Trials. 2021 Nov 24;22:834. doi: 10.1186/s13063-021-05731-6 (PMC8611400; doi:10.1186/s13063-021-05731-6)

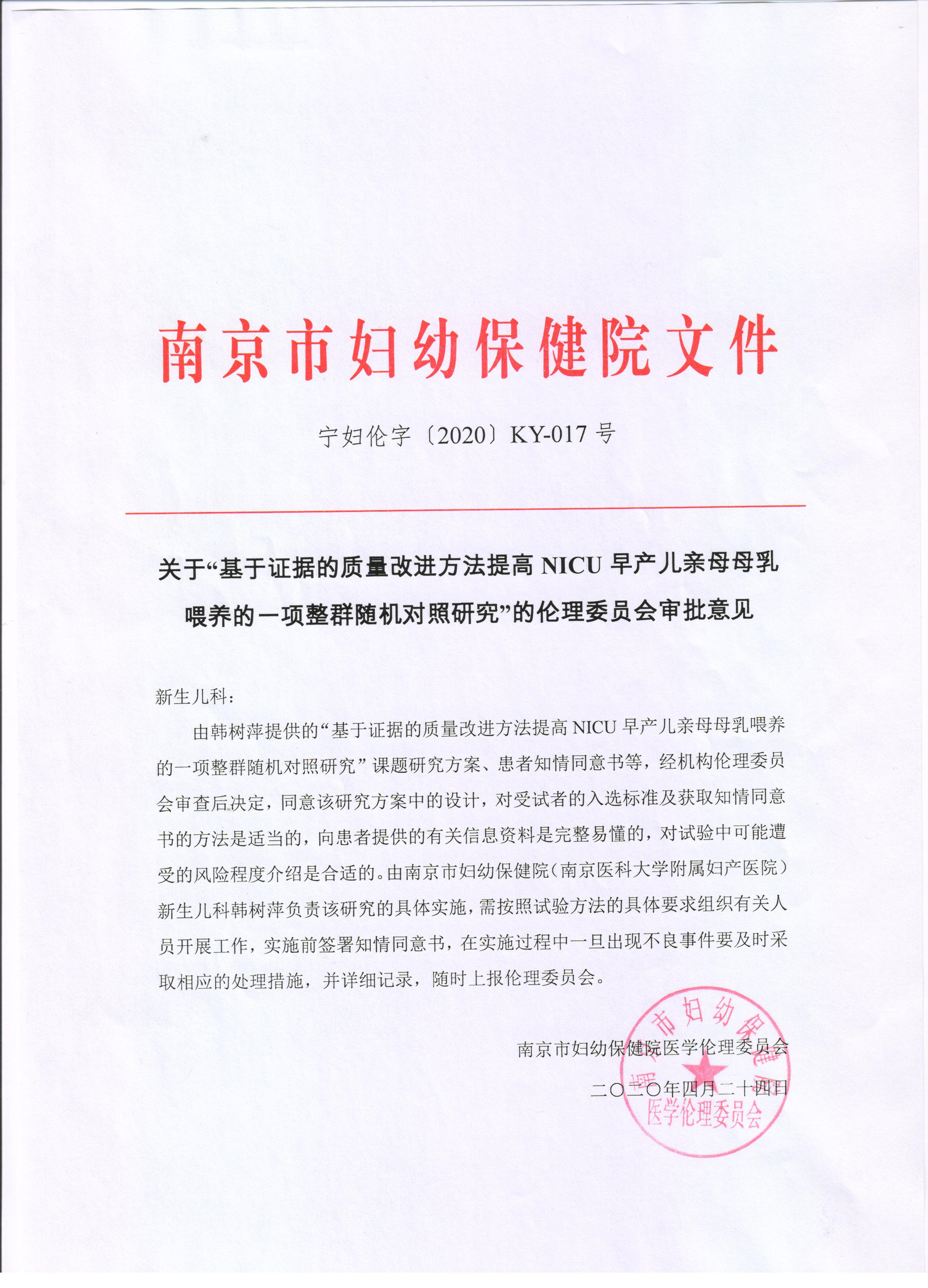

Supplement: Supplementary file 3 — Additional file 3. [file 13063_2021_5731_MOESM3_ESM.jpg]
